# Phenotype of early-onset fetal growth restriction in sheep

**DOI:** 10.3389/fendo.2024.1374897

**Published:** 2024-03-13

**Authors:** Amy E. Sutherland, Tegan A. White, Charmaine R. Rock, Beth R. Piscopo, Ingrid Dudink, Ishmael M. Inocencio, Zahrah Azman, Yen Pham, Ilias Nitsos, Atul Malhotra, Tamara Yawno, Graeme R. Polglase, Graham Jenkin, Emily J. Camm, Beth J. Allison, Suzanne L. Miller

**Affiliations:** ^1^ The Ritchie Centre, Hudson Institute of Medical Research, Melbourne, VIC, Australia; ^2^ Department of Obstetrics and Gynaecology, Monash University, Melbourne, VIC, Australia; ^3^ Department of Paediatrics, Monash University, Melbourne, VIC, Australia; ^4^ Monash Newborn, Monash Children’s Hospital, Melbourne, VIC, Australia

**Keywords:** FGR, IUGR, brain sparing, neurodevelopment, brain injury, asymmetric growth, preterm, postnatal

## Abstract

**Introduction:**

Fetal growth restriction (FGR) is a common pregnancy complication, caused by placental insufficiency, with serious adverse consequences for development *in utero* and postnatal wellbeing. There are no antenatal treatments to improve growth or organ development in FGR, and animal models are essential to mimic the physiological adaptations in FGR and to assess potential interventions. This study aimed to identify the temporal nature of reduced developmental trajectory in fetuses with FGR, and to examine the effects of common factors that may mediate differential growth such as glucocorticoid treatment. We hypothesised that the trajectory of growth would be adversely impacted by FGR.

**Methods:**

FGR was induced via surgical placental insufficiency in fetal sheep (89 days gestation/0.6 gestation; n=135) and compared to age-matched controls over the last third of gestation and into neonatal life (n=153).

**Results:**

Body weight of FGR fetuses/lambs was significantly reduced compared to controls (p<0.0001) from 127 days of gestation (term is 148 days), with increased brain:body weight ratio (p<0.0001) indicative of brain sparing. All biometric measures of body size were reduced in the FGR group with the exception of biparietal (head) diameter. The trajectory of body growth in the last trimester of sheep pregnancy was significantly reduced in the FGR group compared to controls, and stillbirth rate increased with longer gestation.

**Discussion:**

This work provides a well characterised FGR animal model that mimics the known physiological adaptations in human pregnancy and can be used to determine the efficacy of potential interventions.

## Introduction

Fetal growth restriction (FGR) is a complex obstetric condition in which fetal growth is pathologically reduced, most often because the placenta fails to deliver an adequate supply of oxygen and nutrients to support normal fetal development (1, 2). FGR is common, affecting 6-9% of pregnancies in high-resource countries (3, 4). *Placental insufficiency* is the umbrella term used to describe abnormal development and function of the placenta (1), characterised by reduced uteroplacental blood flow, reduced gas and substrate transfer across the placenta, and reduced fetal growth. In response to placental insufficiency, the growth restricted fetus experiences hypoxia (5), initiating a haemodynamic response to preferentially supply essential organs (brain and heart) by redistributing cardiac output (6). In FGR, this adaptive response can be prolonged, which results in cerebrovascular compensation (brain sparing) and asymmetric fetal growth with relatively spared head size but a thinner and/or shorter body (7). A decade ago, the terms FGR or IUGR (intrauterine growth restriction) were often used interchangeably with small for gestational age (SGA). The 2016 consensus definition for FGR has provided an essential framework to delineate infants with pathological FGR, and greater susceptibility for mortality and morbidity, from SGA infants who are constitutionally small but otherwise healthy. SGA is now used to describe any infant <10^th^ percentile for estimated fetal weight or birth weight relative to gestational age and sex, while true FGR is defined as an estimated fetal weight <10^th^ percentile together with antenatal Doppler indices of uteroplacental dysfunction, or estimated fetal weight <3^rd^ percentile as a sole factor (2).

Infants with FGR are often delivered preterm, particularly when the FGR is early-onset (diagnosed at less than 32 weeks’ gestation) (8), and FGR is the strongest risk factor for perinatal death/stillbirth (8, 9). Infants born preterm are likely to be exposed to antenatal glucocorticoids to induce lung maturation, but these glucocorticoids may have differential effects on organ development in FGR and appropriately grown fetuses (10, 11). After birth, FGR is associated with neonatal cardiovascular, respiratory and neurological morbidities at significantly elevated rates compared to appropriate for gestational age infants (7). For example, heart shape and cardiovascular function are altered (12–14) and infants born with FGR spend more time on ventilation and in neonatal intensive care than age-matched appropriately grown infants (15). Despite the presence of brain sparing *in utero*, infants born growth restricted have an increased likelihood of neurodevelopmental deficits in childhood that include poor cognitive function and reduced intelligent quotient (IQ) scores (3, 16, 17) as well as a 10-30-fold increased risk of developing the motor deficit cerebral palsy (18). Determining organ-specific structural and functional changes associated with placental insufficiency and FGR requires appropriate animal models in which major organ development and physiological adaptations replicate the known deficits in human FGR.

There are multiple animal models of placental insufficiency, chronic fetal hypoxia and/or FGR, utilising small and large animal experimental designs (19, 20). A recent systematic review reports that mice and rats were the most used animals for intervention studies in FGR (79%) followed by sheep (16%) (21). The clinical complexity and heterogeneous nature of human FGR means that no single animal model will perfectly mimic the human condition. However, animal models can provide a standardised set of parameters and outcomes allowing the assessment of outcomes of interest. For example, human early-onset FGR is often complicated by very preterm birth which makes it difficult to distinguish the effects of FGR alone on organ development. Animal models can be used to perform separate studies of FGR, very preterm birth, glucocorticoid exposure, and combinations of these factors, such as FGR and very preterm birth (20, 22). Large animal studies of FGR, often in fetal sheep, permit long-term and *in utero* monitoring of the fetus with the addition of vascular catheters and other monitoring techniques (10, 19, 23–27). Subsequently, studies in large animals allow the examination of the fetal physiological response to placental insufficiency and chronic hypoxia, together with the progression of indices of specific organ structural and functional derangement. In turn, therapeutic interventions can be assessed.

The use of a well characterised and clinically useful animal model of FGR is essential for translational studies to improve outcomes for infants born growth restricted. We have been studying FGR in sheep for more than 15 years (28). Herein, we present combined cohort data to describe the phenotype of our model. We utilise a technique called single umbilical artery ligation (SUAL), undertaken during surgery, which causes placental insufficiency via infarction of approximately half of the ovine placental cotyledons as first described in the late 1960’s (29). The SUAL procedure can be carried out at different timepoints in sheep pregnancy, and we have done this to investigate and compare the effects of early-onset FGR (0.6 gestation) versus late-onset FGR (0.72 gestation) on brain development (30). In the work presented here, we include all animals from our early-onset FGR cohort, including singleton pregnancies that we predominantly use to examine the effects of FGR versus control on lambs after birth, and twin pregnancies in which we induce FGR in one fetus, while the second fetus acts as an internal control, used predominantly for fetal studies. We have examined fetal and neonatal lamb outcomes at various ages, allowing us to describe the progression of FGR on organ development. The aim of this study was to characterise the impact of early-onset placental insufficiency on the temporal nature of reduced developmental trajectory in fetuses with FGR, and to examine whether common factors that mediate altered growth (antenatal glucocorticoids, singleton vs twin, fetal sex) differentially affected FGR animals across biometric measures of growth and organ development, compared to gestation-matched control animals.

## Materials and methods

All animal experiments were approved by the Hudson Institute Animal Ethics Committee (approval numbers MMCA 2014/04, MMCA, 2016/19, MMCA 2016/24, MMCA 2016/62, MMCA 2017/38, MMCA 2019/02, MMCA 2019/04, MMCA 2020/06, MMCA 2022/08) and were performed between 2014 and 2023. Animal experiments were conducted as per the National Health and Medical Research Council of Australia Code of Practice for the Care and Use of Animals for Scientific Purposes (Eighth Edition) and comply with the ARRIVE guidelines for reporting animal research (31). Animals had free access to food and water, with food only removed 18 hours prior to surgery.

### Animal preparation

**Figure 1:**
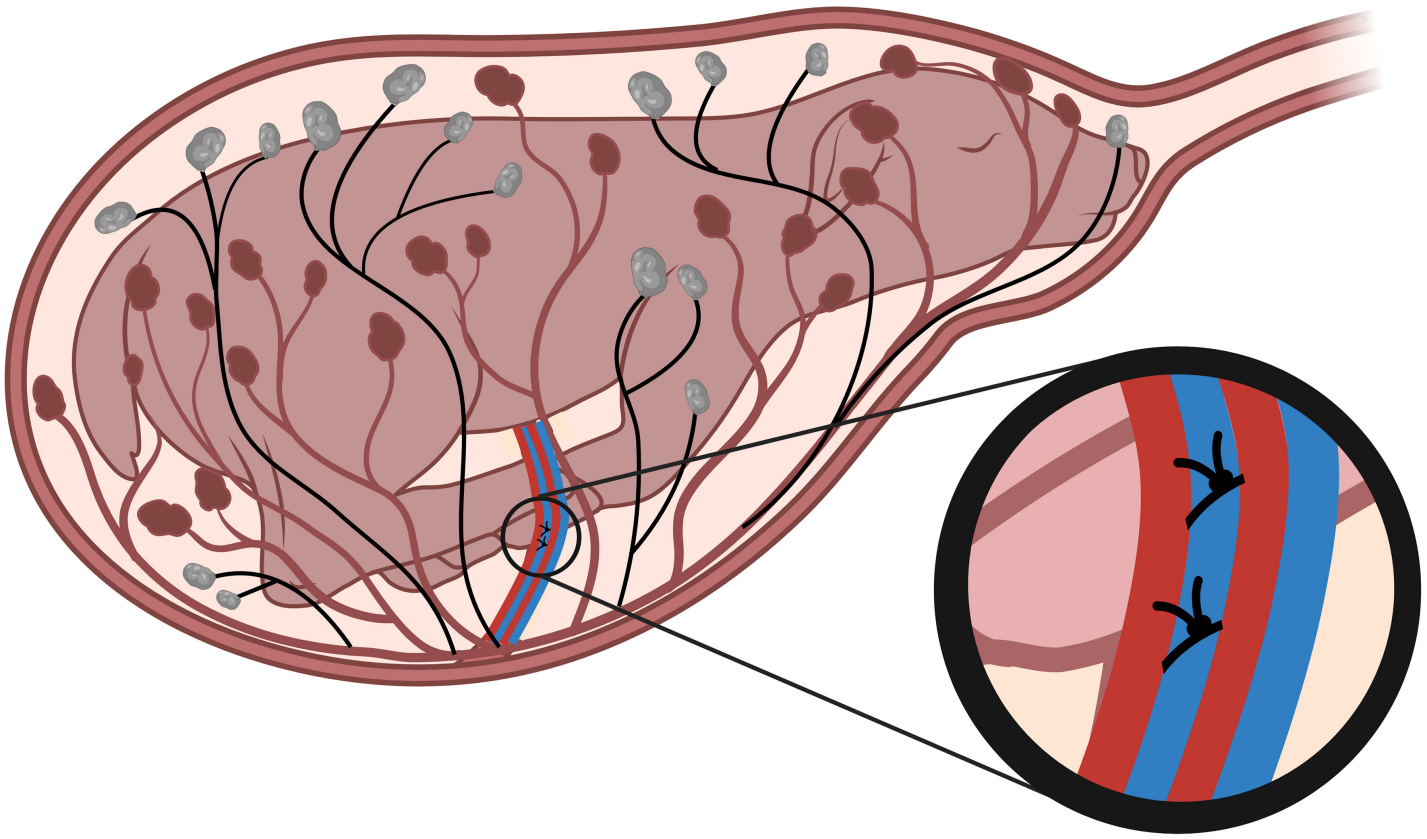
Single umbilical artery ligation. Single umbilical artery ligation (SUAL) surgery is conducted at 88-90 days of gestation (term = 148 days). Two ligatures are secured tightly around one of the two ovine umbilical arteries (blue, inset), resulting in necrosis of approximately half of the placental cotyledons (black). Created with BioRender.com.

Singleton (n=58) or twin-bearing (n=149) Border-Leicester crossbred time-mated pregnant ewes were obtained from Monash Animal Research Platform Gippsland Field Station. On arrival into the Monash Health Translation Precinct Animal Facility, ewes were weighed and this was recorded. At fetal gestational age 88-90 days gestation (0.6 gestation; term is 148 days), surgery was undertaken on the pregnant ewe for induction of placental insufficiency and FGR (30, 32). Briefly, ewes were anaesthetised by injection of sodium thiopentone (Pentothal, 1g in 20mL; Jurox, NSW, Australia) followed by intubation and maintenance of anaesthesia with 1.5–2.5% inhaled isoflurane in O_2_ (Isoflo; Abbott Australasia, Botany, NSW, Australia). An incision was made in the uterus, overlying the fetus. The back legs and torso of the fetus were removed up to the level of the abdomen, and within the umbilical cord the two arteries and veins were identified. A small incision was made in the umbilical cord sheath, approximately 3cm from the fetal abdomen, and one umbilical artery was isolated. Single umbilical artery ligation (SUAL) was undertaken by placing two silk sutures around this artery and tying these tightly (**Figure 1**). In control fetuses, sham-SUAL was performed by isolating and handling the umbilical artery, but with no ligation. In the case of twin-bearing ewes, one fetus was randomly chosen to undergo the SUAL procedure and the other was a sham-SUAL control. A subset of fetuses from twin pregnancies (FGR and control), were instrumented with a femoral artery catheter (inner diameter 0.5mm, outer diameter 1.0mm; Critchley Electrical, NSW, Australia) for blood sampling. Each fetus was returned to the uterus and the uterine incision was closed. A jugular vein catheter was placed in the ewe to allow intravenous antibiotic administration (1g Ampicillin, Aspen Pharmacare Australia, Australia; 500mg Engemycin, Coopers Animal Health, Australia) once daily for the first three days after surgery. The ewes and fetuses were monitored daily to confirm wellbeing. In animals with femoral artery catheters, a fetal blood sample (~200µL) was collected once each day (at approximately 9am) for immediate assessment of blood pH, oxygen saturation (SaO_2_%), partial pressure of oxygen (PaO_2_), partial pressure of carbon dioxide (PaCO_2_), lactate and glucose using an ABL 700 blood gas analyser (Radiometer, Copenhagen, Denmark). Fetal catheters were also flushed again in the afternoon with heparinised saline in an effort to maintain patency of the small fetal femoral artery catheters. In the fetal sheep with post-mortem scheduled for 136-138 days gestation, cord blood was collected from the umbilical vein (~5mL) at the time of post-mortem for assessment of cortisol concentration as per manufacturer’s instructions (ABIN1052524, Blue Gene, Shanghai).

In a subset of ewes, betamethasone was administered as part of their experimental protocol. The ewes received an intramuscular betamethasone injection (11.4mg; Celestone Chronodose, Schering Plough, Australia) 48 hours and 24 hours prior to post-mortem at 127 days gestation. In lambing cohorts, ewes received betamethasone as above, as well as intramuscular mifepristone (50mg; Mifepristone Linepharma, MSHealth, Australia) 48 hours prior to delivery at gestational age 136 days; these lambs were used in the 24 hour and 4 week postnatal cohorts. To ensure the ewe and lamb had an opportunity to bond we did not intervene during delivery unless necessary for animal welfare. Birth weights and measures of lamb well-being were recorded once the ewe had interacted with the lamb and were completed as quickly as possible to minimise the amount of time the lamb was separated from the ewe.

At scheduled post-mortem, ewes and their fetuses or lambs were euthanised with intravenous phenobarbitone (100mg/kg IV, Lethobarb, Virbac Pty Ltd, Peakhurst, Australia) at ~110 days gestation, ~127 days gestation, ~136-138 days gestation, or at 24 hours postnatal age or 4 weeks postnatal age. All fetuses and lambs had biometry measurements, body weights and organ weights recorded at post-mortem.

### Statistical analysis

Data are presented as the mean ± the standard error of the mean (SEM), with the exception of the body weight for the normative growth curve, as undertaken in human presentation of similar data. Blood gas parameters were analysed using a mixed-effects model and postnatal weight gain was analysed using two-way repeated measures ANOVA with time and fetal growth status (FGR or control) as variables, and *post-hoc* Šidák test where appropriate. Body weight and brain/body weight at post-mortem, as well as the effects of fetal sex, betamethasone, and singleton vs twins were analysed using a two-way ANOVA, and *post-hoc* Šidák test where required. Organ weight and biometry comparisons between cohorts (control and FGR) within a gestational age were assessed using t-test. To determine growth trajectory, body weights from separate cohorts collected across gestational ages in control and FGR animals were used to calculate Pearson’s correlation coefficient, and we then compared between groups by analysing whether the coefficient of determination (slope of the lines) between FGR and control groups were different. Statistical comparisons were carried out using GraphPad Prism (GraphPad Software, San Diego, CA, USA, version 9.5). Significance was accepted when p<0.05.

## Results

### Cohort information and fetal arterial blood gas parameters

Data for the number of animals in each group, and survival rates to post-mortem, are presented in **Table 1**. In the 110 days gestation cohort, we include data from n=5 FGR fetuses and 10 control fetuses, with a survival rate for FGR of 63% (5 twins, 3 triplets). In the 127 days gestation cohort we include data from n=98 FGR fetuses and n=105 control fetuses, with a survival rate for FGR of 78% (6 singletons, 121 twins). The 136-138 days gestation cohort includes data from n=4 FGR fetuses and n=9 control fetuses (8 twins). The 24 hours postnatal age cohort includes n=19 FGR lambs and n=17 control lambs (21 singletons, 11 twins), and the 4 week postnatal age cohort includes n=9 FGR lambs and n=12 control lambs (31 singletons, 1 twin). As only 1 death occurred postnatally (1 FGR lamb in 4 weeks postnatal age cohort due to accidental death by mother) we have combined FGR survival rate from the 136-138 days gestation, 24 hours postnatal age and 4 weeks postnatal age cohorts to result in a survival rate for FGR of 62%.

**Table 1:** Success rates for each cohort.

110 days gestation cohort*	Surgery	Survive to post-mortem	Survival rate
Control	N=11	N=10	91%
FGR	N=8	N=5	63%
*Includes 3 surprise triplets where the 3^rd^ fetus was added to the control group			
127 days gestation cohort	Surgery	Survive to post-mortem	Survival rate
Control	N=125	N=105	84%
FGR	N=125	N=98	78%
136-138 days gestation cohort*	Surgery	Survive to post-mortem	Survival rate
Control	N=40	N=38	95%
FGR	N=52	N=32	62%
*Includes 24 hour and 4 week postnatal groups as only 1 FGR postnatal death			

Of the lambs in the postnatal age groups, n=2 control and n=3 FGR lambs in the 24 hours postnatal group required respiratory support in the form of supplemental oxygen immediately after birth (via face mask). All lambs were kept with their mother for the duration of the experimental period however n=4 control and n=9 FGR lambs in the 24 hours postnatal age cohort and n=4 FGR lambs in the 4 weeks postnatal age cohort required bottle feeding in the days following birth. Lambs were given supplemental bottle feeding with milk expressed from the ewe if not consistently gaining weight (measured 4 hourly) within the first 48 hours of life. Bottle feeds were supplemental only, no lamb was exclusively bottle fed. In ewes carrying a single fetus, maternal weight prior to surgery who were assigned to the control or FGR groups was not different (58.1±5.9kg control fetus or 60.7±1.3kg FGR fetus; p=0.41). Our overall survival rate for SUAL-induced FGR at 0.6 gestation in singleton and twin pregnancies combined was 73%. We do however point out that the survival rate of FGR in the older gestation cohort (136-138 days gestation plus lambs) at post-mortem was reduced to 62%, indicative of an increasing risk of stillbirth as gestation progressed in FGR. In total, we present data for n=153 control fetuses/lambs and n=135 FGR fetuses/lambs following early-onset placental insufficiency.

**Figure 2:**
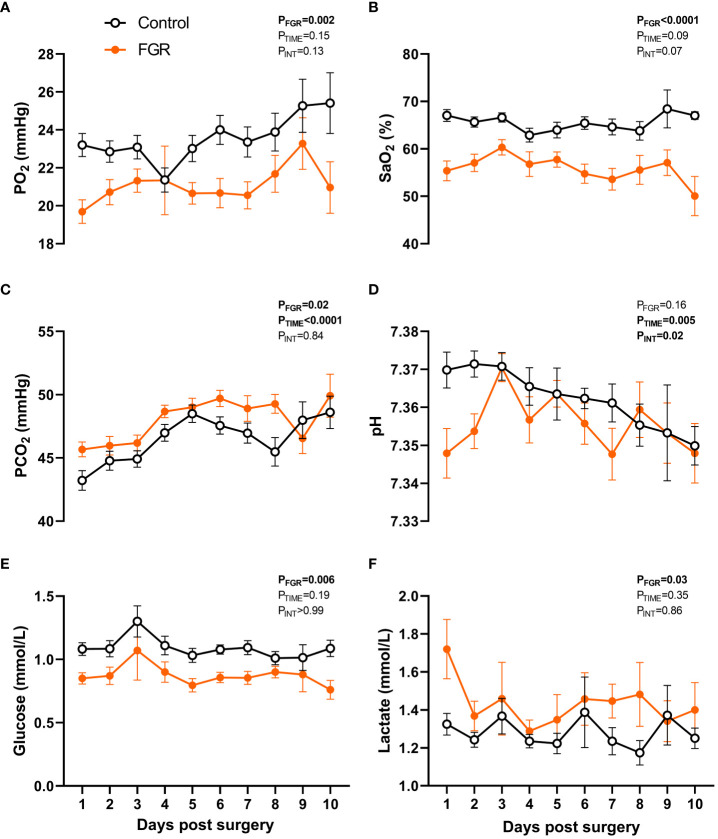
Fetal arterial blood values. Values are mean ± S.E.M. partial pressure of oxygen [PaO_2_; **(A)**], oxygen saturation [SaO_2_; **(B)**], partial pressure of carbon dioxide [PaCO_2_; **(C)**], pH **(D)**, glucose concentration **(E)** and lactate concentration **(F)** in arterial blood samples from fetuses that underwent SUAL at 88-90 days of gestation (FGR, orange) and controls (black) (mixed effects model). Group sample size ranged between n=7-33 for controls and n=5-35 for FGR fetuses.

In fetuses that were instrumented with an arterial catheter (n=36 FGR and n=36 control), blood samples were taken daily between day 1 to 10 after surgery and arterial blood gas parameters assessed (**Figure 2**). Two-way repeated measures ANOVA showed that FGR was associated with reduced circulating partial pressure of oxygen (PaO_2_; p=0.002; **Figure 2A**), reduced oxygen saturation (SaO_2_; p<0.0001; **Figure 2B**), increased partial pressure of carbon dioxide (PaCO_2_; p<0.0001; **Figure 2C**), reduced glucose concentration (p=0.006; **Figure 2E**), and elevated lactate (p=0.033; **Figure 2F**). There was no effect of FGR on pH (p=0.16; **Figure 2D**). We also observed a gestational age-related increase in PaCO_2_ (p<0.0001) and decrease in pH (p=0.005) over the course of the study, reflecting an overall demise in placental function as gestation progressed. Fetal cortisol concentrations were also analysed in a small subset of umbilical cord blood collected from control (n=4) and FGR (n=4) fetuses from the 136-138 days gestation cohort. Compared to control fetuses, circulating levels of cortisol tended to be elevated in FGR fetuses (88.3±17.3ng/mL vs 53.4±8.3ng/mL) but this was not statistically significant (p=0.12; intra assay coefficient of variance 5.3%).

### Body weights

**Figure 3:**
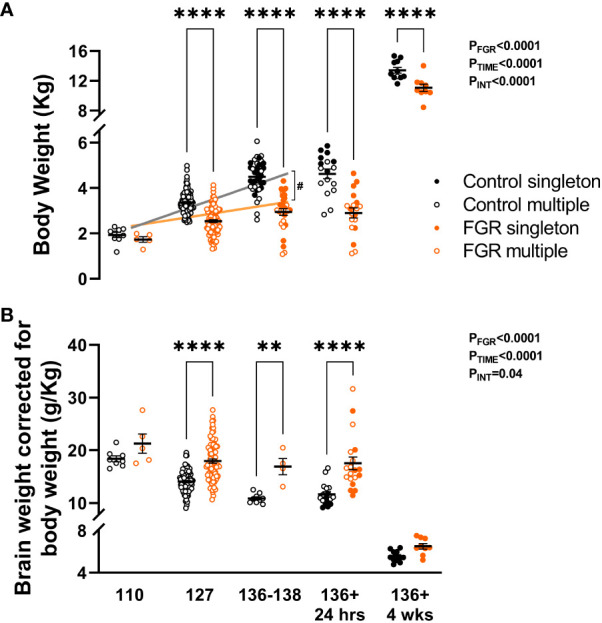
Post-mortem weight. Values are mean ± S.E.M. body weight **(A)** and brain weight adjusted for body weight **(B)** in fetuses and lambs that underwent SUAL at 88-90 days of gestation (FGR singletons, solid orange; FGR twins, open orange) and controls (singletons, solid black; twins, open black). ****p<0.0001, **p<0.01, significant difference between groups (two-way ANOVA + Šídák’s test) with linear regression analysis of fetal cohorts (FGR orange, control grey) #p<0.0001 between groups.

All body weights of control and FGR cohorts were recorded at post-mortem. Two-way ANOVA showed that body weight increased significantly across the ages assessed (p<0.0001) but was significantly reduced in FGR animals compared to controls (p<0.0001; **Figure 3A**). Within age groups, FGR animals weighed less at 127 days gestation (p<0.001), 136-138 days gestation (p<0.001), 24 hours postnatal age (p<0.001) and at 4 weeks postnatal age (p<0.001). Brain weight corrected for body weight was significantly reduced over time (p<0.0001; **Figure 3B**) but was significantly increased in FGR animals compared to control groups (p<0.0001). Within age groups, FGR animals had significantly increased brain to body weight ratio at 127 days gestation (p<0.001), 136 days gestation (p=0.005) and 24 hours postnatal age (p<0.001). Within the control group only, multiple regression analysis showed that body weight was significantly increased from 110 to 127 days gestation (p<0.0001) and between 127 to 136-138 days gestation (p<0.0001). Conversely, within the FGR group body weight did not increase between 110 and 127 days gestation (p=0.44), nor did it increase between 127 and 136-138 days gestation (p=0.22). Linear regression showed a significant upward trajectory of body weight in the control fetal groups (r^2^ = 0.56, p<0.0001) and in the FGR group (r^2^ = 0.13, p<0.0001). The slope of the lines was significantly different (p<0.0001) when comparing the control and FGR group, indicating that overall growth trajectory was adversely impacted in the FGR cohort.

### The effects of fetal sex, betamethasone and singleton vs twins

**Figure 4:**
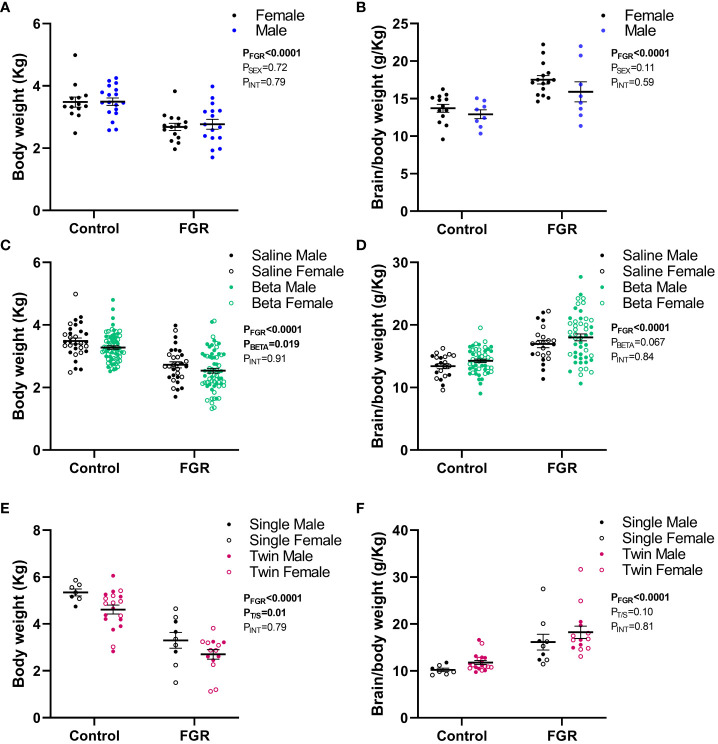
Effect of sex, betamethasone and number of fetuses on post-mortem weight. Values are mean ± S.E.M. body weight **(A)** and brain weight corrected for body weight **(B)** from female (black) and male (blue) fetuses at 127 days of gestation, body weight **(C)** and brain weight corrected for body weight **(D)** in fetuses that received saline (black, male closed circles, female open circles) or betamethasone (green, male closed circles, female open circles) at 127 days of gestation and body weight **(E)** and brain weight corrected for body weight **(F)** in singleton (black, male closed circles, female open circles) and twin (pink, male closed circles, female open circles) fetuses at 136-138 days of gestation and lambs at 24 hours postnatal age that underwent SUAL at 88-90 days of gestation (FGR) and controls (two-way ANOVA).

The effects of male vs female sex and maternal betamethasone administration was investigated in fetuses at 127 days gestation. Using a two-way ANOVA, we did not observe an effect of fetal sex on body weight (p=0.72; **Figure 4A**) or brain weight corrected for body weight (p=0.11; **Figure 4B**). Betamethasone administration significantly reduced body weight (p=0.019; **Figure 4C**) but did not significantly alter brain weight corrected for body weight (p=0.067; **Figure 4D**).

In the 136-138 days gestation and 24 hours postnatal age cohort, we investigated the effect of singleton versus twin pregnancy. Two-way ANOVA revealed that twin pregnancies were associated with a significant reduction in fetal body weight in both control and FGR fetuses compared to singleton pregnancies (p=0.01; **Figure 4E**). Twin pregnancy did not alter brain weight corrected for body weight (p=0.10; **Figure 4F**).

**Figure 5:**
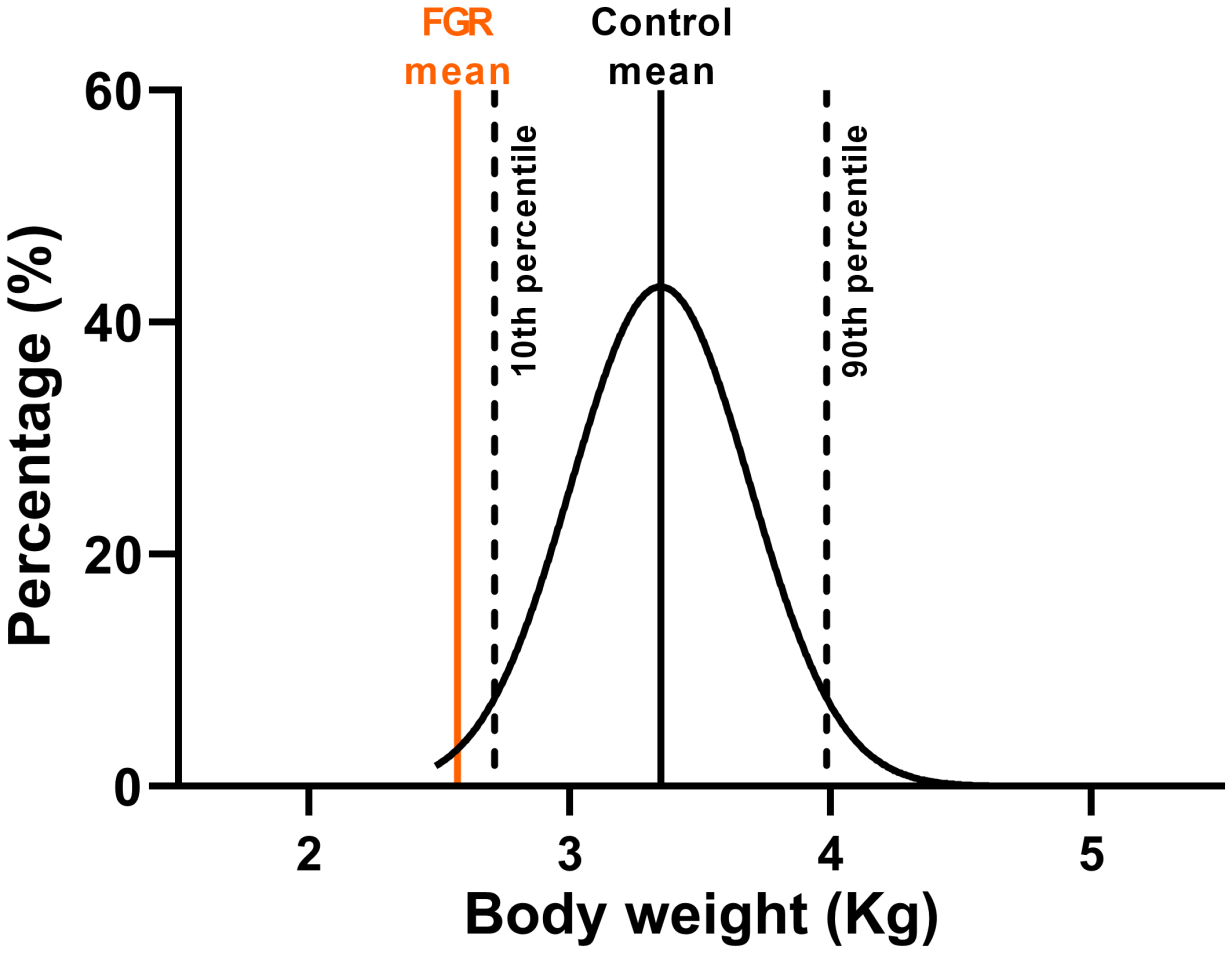
Ovine growth normative curve. Normal distribution of body weight from control fetuses (black) at 127 days of gestation from twin pregnancies with mean FGR body weight at 127 days of gestation (orange).

From **Figure 3**, it is evident that the largest dataset available is from the animals with post-mortem at 127 days gestation (n=105 control and n=98 FGR fetuses). Accordingly, we used this data to create our normative distribution of body weight in our control fetal sheep (**Figure 5**), with a mean body weight of 3.35±0.50kg, and for which we marked the 10^th^ (2.71kg) and 90^th^ (3.99kg) percentiles for normal body weight distribution. The mean body weight of FGR fetuses at this gestational age was 2.54±0.06kg, which sits between the 5^th^-6^th^ percentile on the normative growth chart.

**Figure 6:**
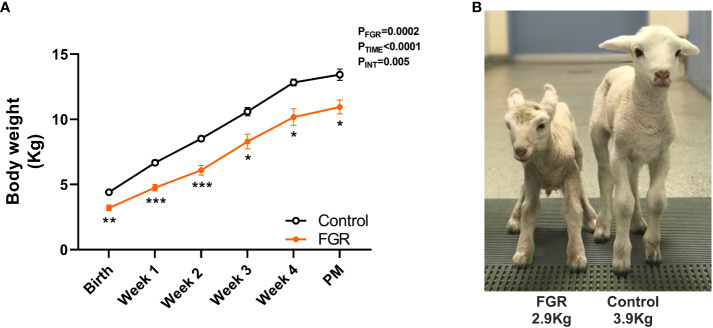
Postnatal growth. Values are mean ± S.E.M. body weight **(A)** in lambs that underwent SUAL at 88-90 days gestation and were born at 136 days of gestation with post-mortem (PM) at 1 month postnatal age (FGR, orange; n=9) and controls (black, n=10). ***p<0.001, **p<0.01, *p<0.05 significant difference between groups (two-way repeated measures ANOVA + Šídák’s test). Image of FGR and control lamb at 24 hours postnatal age **(B)**. Linear regression analysis revealed the slope of the lines were not different between control and FGR lambs (p=0.056).

In lambs followed up to 4 weeks of age (**Figure 6**), body weight was recorded at the time of birth and then weekly until post-mortem. Two-way ANOVA revealed a significant increase in body weight over time (p<0.0001) in both groups, but the weight of the FGR lambs was significantly lower compared to control lambs (p=0.0002). *Post-hoc* analysis shows that compared to control lambs, body weight of FGR lambs was significantly lower at birth (p=0.002), week 1 (p=0.0001), week 2 (p=0.0004), week 3 (p=0.02), week 4 (p=0.02) and at post-mortem (PM; p=0.01). Linear regression analysis showed that there was no difference in the trajectory of postnatal growth between the control and FGR cohorts (p=0.06).

### Biometry and organ weights

In the 127 days gestation group, we examined the effects of placental insufficiency and FGR on key organ weights in growth restricted and control cohorts (**Table 2**) recorded at post-mortem. After adjustment for body weight at 127 days (3.36±0.05kg control vs 2.54±0.06kg FGR; p<0.0001), brain weight, adrenal weight, and kidney weight were all significantly increased in the FGR offspring (all p<0.0001). The ratio of heart, lung, liver and spleen weight to body weight were not different in control and FGR groups (p>0.05). Similarly, in the 127 days gestation group we recorded measures of fetal biometry (**Table 2**) – crown rump length, tibia length, femur length, lower limb length, abdominal circumference and biparietal diameter. Results showed that crown-rump length, abdominal circumference, femur length, tibia length and lower leg length were significantly reduced in the FGR animals compared to the control group (p<0.01). In this same cohort, we made qualitative observations on the gross morphology of placental cotyledons from control and FGR offspring. The placental cotyledons from control fetuses were almost always healthy in appearance, with a predominance of type B placentomes (33), while more than 50% of placentomes associated with FGR offspring were necrotic and remaining placentomes showed a predominance of type C flattened shape and to a lesser extent type D morphology.

**Table 2:** Body and organ weights, and biometric measures of growth from 127 day cohort.

	Control N=69-89	FGR N=69-86
**Body weight (Kg)**	3.36 ± 0.05	2.54 ± 0.06****
**Brain: body weight (g/Kg)**	14.07 ± 0.22	17.97 ± 0.40****
**Heart: body weight (g/Kg)**	8.32 ± 0.18	8.17 ± 0.21
**Adrenal: body weight (g/Kg)**	0.12 ± 0.00	0.15 ± 0.00****
**Lung: body weight (g/Kg)**	30.07 ± 0.63	29.08 ± 0.90
**Kidney: body weight (g/Kg)**	7.01 ± 0.16	7.81 ± 0.16***
**Liver: body weight (g/Kg)**	31.82 ± 0.65	30.42 ± 0.70
**Spleen: body weight (g/Kg)**	1.46 ± 0.05	1.36 ± 0.05
**Crown rump length (cm)**	43.97 ± 0.43	40.05 ± 0.43****
**Biparietal diameter (cm)**	8.90 ± 0.26	8.48 ± 0.27
**Abdominal circumference (cm)**	32.45 ± 0.30	29.34 ± 0.41****
**Femur length (cm)**	11.88 ± 0.20	10.63 ± 0.20****
**Tibia length (cm)**	14.60 ± 0.23	13.29 ± 0.21****
**Lower leg length (cm)**	13.95 ± 0.28	12.91 ± 0.27**

****p<0.0001, ***p<0.001, **p<0.01 compared to control.

## Discussion

Experimental models of placental insufficiency and FGR are critical tools to advance our understanding of the physiology of altered growth in FGR fetuses, and for the translation of new therapies to improve outcomes. Animal models of FGR allow characterisation of the fetal response to placental insufficiency, the ontogeny of growth deficits, and cellular mechanisms of organ-specific impairments that are associated with FGR. In the current study, we present novel results to show the temporal progression of reduced body growth in FGR during late gestation in response to early-onset placental insufficiency induced by single umbilical artery ligation (SUAL). In human pregnancies, placental insufficiency may be caused by maldevelopment of the placenta or secondary damage to a normal placenta, but with the common features of reduced oxygen and nutrient transfer (1). In the current sheep study we replicated fetal hypoxaemia and hypoglycaemia relative to control fetuses, and redistribution of fetal combined cardiac output to favour the brain. In human infants, an estimated fetal weight (EFW) less than the 3^rd^ percentile relative to growth charts is described as ‘severe’ FGR (2, 34). The large number of samples available in the current ovine study permitted us to plot a normal ovine birthweight chart, and to determine that the mean body weight for our cohort of FGR sheep fetuses is between the 5^th^–6^th^ percentile on the normal growth chart, thus representing a moderate to severe degree of fetal growth restriction. We assessed multiple time points for separate cohorts of animals for data collection over the final third of gestation and then into the neonatal period, and we show that fetal growth trajectory is significantly reduced in response to placental insufficiency and FGR.

Placental insufficiency is the most well recognised cause of FGR (1) and uteroplacental dysfunction is now incorporated into the diagnostic criteria for clinical FGR through altered Doppler flow patterns (2). We adopted the model of SUAL because it induces placental insufficiency, causing gross and microscopic evidence of placental infarction in about half of the ovine placental cotyledons (29). The surgery to undertake SUAL can be conducted across a range of gestational ages, and previously we have compared the effects of early-onset and late-onset placental insufficiency and FGR on fetal brain development (30). All animals in the data presented in this study were from the early-onset cohort. Over the duration of fetal blood sample collection, arterial oxygenation was reduced by 15% compared to control, and glucose was 20% lower. This is in good agreement with mild chronic hypoxaemia observed in human infants when cord blood was sampled at birth (35). It is well described that *in utero* fetal hypoxaemia induces an adaptive cardiovascular response to redistribute cardiac output to essential organs, notably the brain, and hence this response is often called brain sparing (6). In the current study, fetal hypoxaemia was present within the first week after the onset of placental insufficiency relative to the controls, and while fetal body weight was not significantly reduced at 110 days gestation (>20 days after onset of placental insufficiency), body weight was reduced at 127 days gestation. Notably, in the control group there was a significant increase in weight gain between 110 to 127 days gestation, and again between 127 to 136-138 days, indicative of the positive trajectory of normal fetal growth during late gestation. In the FGR cohort, there was no significant increase in fetal weight between either 110 to 127 days, or 127 to 136-138 days gestation, confirming that the growth trajectory of FGR fetuses was severely impacted. We also examined the effects of perinatal factors on gross body weight in control and FGR offspring and found that fetal sex was not associated with altered growth, however both antenatal glucocorticoid (betamethasone) administration and being a twin were associated with reduced growth in both control and FGR fetuses. Previous studies in fetal sheep have shown growth restriction with antenatal glucocorticoid exposure (36), which is an effect also seen in human infants with a single course of antenatal glucocorticoids who then deliver at term (37). In the current study the degree of growth restriction induced by betamethasone was similar in control and FGR offspring, with no interaction between factors.

Prolonged redistribution of cardiac output in response to chronic fetal hypoxia produces asymmetric fetal growth, via the brain sparing response. This adaptive haemodynamic response is caused by local vasodilatation of the fetal cerebral vascular bed and a sustained increase in fetal peripheral vascular resistance and bradycardia that are mediated by carotid chemoreflexes (6). In the clinical situation, this response to fetal hypoxia is detected as a change in cerebral perfusion using Doppler ultrasound, predominantly via a vasodilatation of the middle cerebral artery (MCA) with evidence of reduced pulsatility index (PI) (38). A decreased MCA PI is correlated with worsening fetal hypoxaemia (39) and increasing incidence of brain injury (40–42). Ultrasound measures of fetal biometry are also used to diagnose FGR, most commonly head circumference, abdominal circumference, biparietal diameter and femur length (43). In this sheep study, we were able to replicate multiple biometric measures to demonstrate reduced fetal growth, including significantly reduced birth weight, reduced abdominal circumference and reduced femur length. Brain to body weight ratio was significantly increased, demonstrating asymmetric growth restriction and brain sparing. Lung, liver and spleen weight tended to be lower with FGR, these were not significant to control values, and we also observed that adrenal and kidney weight to body weight ratios were significantly increased, indicating preferential sparing of these organs in the FGR cohort. While brain sparing was apparent in this cohort of FGR offspring, our studies to date demonstrate that the brain is not spared from injury (22, 32, 44–47). We are however mindful that the peak in perinatal brain growth occurs earlier in sheep than in humans (48), in part we accounted for this by inducing early lamb delivery, but future studies should match brain function and structure outcomes in lambs (49, 50) to more comprehensively determine the consequences of early-onset FGR on longer term neurodevelopmental outcomes.

Across the gestational ages studied in this ovine model of FGR, we report the survival rate for the FGR procedure. Overall, this figure is 73% survival of FGR fetuses/lambs to scheduled post-mortem. When examined more closely, we noted a 62% survival rate in the late gestation cohort. This is not surprising, given that it is well described clinically that FGR is the strongest risk factor for stillbirth/perinatal death (8, 9). Two clinical trials have evaluated the effects of the timing of delivery in infants with early-onset FGR (the GRIT and TRUFFLE trials) (51). Both studies were designed to compare immediate delivery versus delayed delivery guided by fetal cardiovascular monitoring, and while they were not powered to assess the effects of gestational age at delivery, results show an increased likelihood of fetal or neonatal death in the delayed delivery cohort (51). This is supported by population data to show that FGR is a significant risk factor for late preterm stillbirth, with undetected FGR linked to a 5-fold increased risk of stillbirth (9). Future studies could utilise this ovine model of FGR to elucidate whether there are physiological signs or biomarkers in the growth restricted fetus prior to fetal demise.

Early-onset FGR, diagnosed prior to 32 weeks fetal gestational age, is the more severe form of fetal growth restriction, associated with preterm birth, significant fetal hypoxia and cardiovascular adaptation (5). Early-onset FGR occurs less commonly than late-onset FGR (early-onset comprises 20-30% of FGR cases), but is linked to high rates of perinatal mortality, and high likelihood of both neonatal and long-term morbidities of respiratory control, the cardiovascular system, and neurological function (7). Clinical trials to reduce mortality and morbidity in this cohort are extremely limited, but one trial was the international STRIDER trial – Sildenafil therapy in dismal prognosis early-onset fetal growth restriction – which hypothesised that maternal sildenafil citrate treatment in early-onset FGR would improve perinatal wellbeing via increased uteroplacental perfusion with sildenafil administration (52). Unfortunately, the STRIDER trial was halted prematurely due to an indication in the Dutch STRIDER study that sildenafil may have been associated with an increased rate of neonatal death (53). This finding supports the strong need to firstly assess the effects of treatments in well characterised animal models of FGR (23, 54, 55).

The first documented studies of SUAL were undertaken by Emmanouilides in 1968 (29), subsequent studies have added information on the late-onset model (56, 57), reporting mixed success, particularly when the SUAL procedure occurs later. Over time in our studies, we have found that early-onset SUAL in pregnant sheep (before 100 days gestation) is much more reliable at producing FGR than late onset (after 105 days gestation), with a better survival rate of >70%. Over time, we have also noted differential effects of analgesia in this animal model; when we commenced this work our analgesic of choice for sheep studies was fentanyl, however fentanyl itself has effects on fetal haemodynamics, including tachycardia and cerebral vasodilation (58) and we noted high rates of FGR fetal death that we surmised were due to fentanyl interfering with the brain sparing cardiovascular response that follows placental insufficiency. All ewes are now given oral or rectal paracetamol for pain relief. A limitation of the current study was that we did not collect and record weights or perform morphometric analysis on the placental cotyledons. Anecdotally, we observed that >50% of placental cotyledons from FGR offspring were necrotic and remaining healthy placentomes were more likely to be a type C with a flattened morphology that is associated with intrauterine hypoxia (33). This study provides a large overall sample size of n=135 FGR offspring and n=153 control offspring, however we acknowledge that these animals are derived from multiple studies and therefore some outcome measures were not available for all animals; for example there is a relatively small cohort of animals with fetal blood gas parameters and these were difficult to maintain during chronic recordings. Thus, a novelty of this work is that we compared control and FGR animals at various fetal and neonatal timepoints, but the experimental design means that individual fetuses were not tracked over time, rather the results of multiple animals were grouped.

In summary, we induced early-onset FGR at 88-90 days (0.6) of sheep gestation and measured physical characteristics and organ development compared with control offspring over the last trimester of ovine pregnancy and into neonatal life. Our results clearly define the phenotype of FGR in this model, with chronic fetal hypoxaemia apparent *in utero*, and no difference in fetal weight between the FGR and control groups at 110 days gestation but then significant growth restriction thereafter as gestation progresses. We observed a significant upward trajectory of growth in the control fetuses during the last trimester of pregnancy, but the trajectory of fetal development was significantly reduced in the FGR cohort. Finally, this ovine model of FGR is associated with stillbirth as gestation progresses, which may provide a novel animal model to assess the fetal physiological responses associated with fetal deterioration. The use of preclinical animal models of pregnancy and birth compromise allows specific characterisation of how structural deficits underpin functional deficits of multiple organ systems including the brain, heart and lungs (27, 32, 50, 59–61). In animal experiments, the wellbeing of the fetus can be monitored *in utero*, postnatal clinically applicable assessments such as MRI and haemodynamic monitoring can be performed (62–64), and these can all be correlated with cellular level assessment of structural alterations in organs collected at post-mortem (64, 65). Such studies to reveal fundamental relationships between organ structure and function are essential to better our understanding of how fetal compromise alters the trajectory of development, and these studies cannot be undertaken in humans. Well characterised animal models of placental insufficiency and FGR are also critical for examination of therapeutics to target improved growth, organ development and function.

## Data availability statement

The raw data supporting the conclusions of this article will be made available by the authors, without undue reservation.

## Ethics statement

The animal study was approved by Hudson Institute of Medical Research Animal Ethics Committee. The study was conducted in accordance with the local legislation and institutional requirements.

## Author contributions

AS: Data curation, Formal analysis, Investigation, Project administration, Visualization, Writing – original draft, Writing – review & editing. TW: Investigation, Writing – original draft, Writing – review & editing. CR: Investigation, Writing – original draft, Writing – review & editing. BP: Investigation, Writing – original draft, Writing – review & editing. ID: Investigation, Writing – original draft, Writing – review & editing. II: Investigation, Writing – original draft, Writing – review & editing. ZA: Investigation, Writing – original draft, Writing – review & editing. YP: Investigation, Writing – original draft, Writing – review & editing. IN: Investigation, Writing – original draft, Writing – review & editing. AM: Investigation, Writing – original draft, Writing – review & editing. TY: Investigation, Writing – original draft, Writing – review & editing. GP: Investigation, Funding acquisition, Writing – original draft, Writing – review & editing. GJ: Investigation, Writing – original draft, Writing – review & editing. EC: Investigation, Writing – original draft, Writing – review & editing. BA: Conceptualization, Formal analysis, Investigation, Funding acquisition, Project administration, Methodology, Supervision, Visualization, Writing – original draft, Writing – review & editing. SM: Conceptualization, Formal analysis, Investigation, Funding acquisition, Project administration, Methodology, Supervision, Visualization, Writing – original draft, Writing – review & editing.
